# Dendritic stratification differs among retinal OFF bipolar cell types in the absence of rod photoreceptors

**DOI:** 10.1371/journal.pone.0173455

**Published:** 2017-03-03

**Authors:** Christian Puller, Patrick Arbogast, Patrick W. Keeley, Benjamin E. Reese, Silke Haverkamp

**Affiliations:** 1 Department of Neuroscience, Carl von Ossietzky University, Oldenburg, Germany; 2 Max Planck Institute for Brain Research, Frankfurt am Main, Germany; 3 Neuroscience Research Institute, University of California Santa Barbara, Santa Barbara, California, United States of America; 4 Department of Psychological and Brain Sciences, University of California Santa Barbara, Santa Barbara, California, United States of America; 5 Institute of Cellular and Molecular Anatomy, Goethe-University, Frankfurt am Main, Germany; University of Florida, UNITED STATES

## Abstract

Retinal OFF bipolar cells show distinct connectivity patterns with photoreceptors in the wild-type mouse retina. Some types are cone-specific while others penetrate further through the outer plexiform layer (OPL) to contact rods in addition to cones. To explore dendritic stratification of OFF bipolar cells in the absence of rods, we made use of the ‘cone-full’ *Nrl*^*-/-*^ mouse retina in which all photoreceptor precursor cells commit to a cone fate including those which would have become rods in wild-type retinas. The dendritic distribution of OFF bipolar cell types was investigated by confocal and electron microscopic imaging of immunolabeled tissue sections. The cells’ dendrites formed basal contacts with cone terminals and expressed the corresponding glutamate receptor subunits at those sites, indicating putative synapses. All of the four analyzed cell populations showed distinctive patterns of vertical dendritic invasion through the OPL. This disparate behavior of dendritic extension in an environment containing only cone terminals demonstrates type-dependent specificity for dendritic outgrowth in OFF bipolar cells: rod terminals are not required for inducing dendritic extension into distal areas of the OPL.

## Introduction

Retinal bipolar cells are a diverse class of excitatory interneurons, passing the photoreceptor signals on to amacrine and ganglion cells (reviewed in [1]). ON bipolar cells depolarize, while OFF bipolar cells hyperpolarize in response to light increments. The different types of OFF bipolar cells have long been thought to contact cone photoreceptors only, unselectively via the flat contacts of their dendritic tips with the cone pedicle base [2–4]. However, more recent studies have shown that this assumption of cone-specific wiring of OFF bipolar cells is inaccurate.

Substantial evidence has accumulated for OFF bipolar cells contacting rods as well as cones in different mammalian species [5–13]. OFF bipolar cells are, consequentially, now considered to provide a tertiary rod signaling pathway in addition to the rod ON bipolar cell and the electrical coupling of rods to cones via gap junctions. Interestingly, contacts between OFF bipolar cells and rods occur in a type-dependent manner. Among the five types of mouse OFF bipolar cells [14], rod contacts are formed by only three types (3a, 3b, 4), while types 1 and 2 appear to be cone selective [5, 6, 15]. The disparate behavior in photoreceptor connectivity suggests that rod contacts may be selectively specified as cone bipolar cell dendrites seek their synaptic partners.

It remains unclear, however, if the presence of rods is required to elicit dendritic outgrowth into more distal areas of the OPL, where rod terminals are typically located in wild-type retinas. The neural retina leucine zipper knockout mouse (*Nrl*^*-/-*^) is an excellent model to explore dendritic patterning of OFF bipolar cells in this regard. The *Nrl* gene encodes a transcription factor which in turn regulates several genes required for rod development from photoreceptor precursor cells [16–19]. Nrl therefore acts as a molecular switch which, if turned off, forces all photoreceptor precursors into a cone fate and enhances the expression of short-wavelength sensitive (S-) opsin. Accordingly, photoreceptors of the *Nrl*^*-/-*^ mouse have been shown to consist of only cone-like photoreceptors. Despite the formation of ‘rosettes’ in the outer nuclear layer and some other morphological abnormalities at the cellular level, cone-like photoreceptors in the *Nrl*^*-/-*^ mouse are fully functional and co-express varying ratios of middle-wavelength sensitive (M-) and S-opsins [20, 21]. The *Nrl*^*-/-*^ mouse has therefore been used previously as a mouse model for a ‘cone-full’ retina in the absence of rods [22–24]. The present study has used this *Nrl*^*-/-*^ mouse model to eliminate a putative extrinsic signal dictating the different wiring patterns of OFF bipolar cells.

Here, we performed an anatomical analysis of OFF bipolar cell types in the *Nrl*^*-/-*^ mouse retina to investigate their dendritic stratification patterns in the absence of rods. Despite their absence, we still found distinct patterns of dendritic stratification between most OFF bipolar cell types. Rod terminals, therefore, are not critical for eliciting dendritic extension of the OFF bipolar cell types that contact rods in wild-type retinas [6, 15].

## Materials and methods

### Tissue preparation

All procedures were approved by the University of California at Santa Barbara Institutional Animal Care and Use Committee (IACUC). These were in full accordance with institutional guidelines of the Max-Planck-Institute for Brain Research, Frankfurt, following the standards described by the German animal protection law (*Tierschutzgesetz*). Euthanasia of mice for organ harvesting (retinas) used in this study has been approved by the animal welfare officer of the Max-Planck-Institute for Brain Research and reported to the local authorities (*Regierungspräsidium Darmstadt*).

Adult mice (*Nrl*^+/+^ and *Nrl*^-/-^, on a C57BL/6J genetic background) were injected with a lethal dose of sodium pentobarbital (120mg/kg). The eyes were dissected, the cornea and lens were removed and the remaining eyecups were then fixed in 4% paraformaldehyde in 0.1 M phosphate buffer (PB), pH 7.4, for 30 min for light microscopy (LM) or 60 min for electron microscopy (EM). After fixation, each retina was dissected from the eyecup and cryoprotected in graded sucrose solutions (10, 20, and 30%). For light microscopy, the retina was embedded in freezing medium (Leica, Nussloch, Germany). Vertical sections of 14 μm thickness were cut on a cryostat (CM 30505, Leica), collected on glass slides, and stored at -20°C. For electron microscopy, the retina was embedded in 4% agar after freezing and thawing the tissue. The agar block was mounted on a vibratome (VT 1000 S, Leica), and vertical sections of 60 μm thickness were cut. Vibratome sections were processed free-floating in 0.01 M phosphate buffered saline (PBS), pH 7.4.

### Immunohistochemistry

The retinal sections were incubated for 1 hour in blocking solution (10% normal donkey serum, 0.5% Triton in PBS) and afterward in a solution containing the primary antibodies (diluted in 3% normal donkey serum, 0.5% Triton in PBS) overnight at room temperature. After extensive washing in PBS, sections were incubated with secondary donkey antibodies conjugated either to Cy3 or Cy5 (Dianova), or Alexa Fluor 488 (Molecular Probes) for 1 hour at room temperature. Bipolar cells were labeled with antibodies against NK3R (rabbit, polyclonal, 1:500; A. Hirano, University of Los Angeles, Los Angeles, USA) [25], HCN4 (rabbit, polyclonal, 1:500; Alomone Labs, APC-052) [8], PKARIIβ (mouse, monoclonal, 1:3000; BD Biosciences, 610625) [8], calsenilin (Csen, mouse, monoclonal, 1:2000; W. Wasco, Harvard Medical School, Charlestown, USA [5], now Millipore, 05–756). Synaptic ribbons were labeled with antibodies against the cytomatrix protein bassoon (mouse, monoclonal, 1:5000; Stressgen Biotechnologies, now Enzo Life Sciences, ADI-VAM-PS003) in case of double-labeling experiments with NK3R in wild-type retina [26]. Antibodies against C-terminal-binding protein 2 (CtBP2, rabbit, polyclonal, 1:5000; Synaptic Systems, Germany, 193 003; and mouse, monoclonal, 1:5000; BD Biosciences, 612044) were applied for the same purpose in all other experiments [27, 28]. Antibodies against GluA1 (rabbit, polyclonal, 1:100; Millipore, AB1504) and GluK1 (goat, polyclonal, 1:100, Santa Cruz Biotechnology, sc-7616) were used to label the corresponding glutamate receptor subunits [29]. Confocal images were taken using either a Zeiss LSM 5 Pascal or an Olympus FV1000 confocal microscope, both equipped with argon and HeNe lasers. High-resolution scanning of image stacks was performed with a Plan-Apochromat 63x/1.40 (Zeiss) or a UPlanSApo 60x/1.35 (Olympus) oil immersion objective using a z-axis increment of 0.25–0.3 μm. Final images were adjusted for brightness and contrast using Photoshop CS5 (Adobe Systems).

### Image analysis and quantification

To quantify the vertical penetration of the OPL by bipolar cell dendrites in the *Nrl*^-/-^mouse, stacks of confocal micrographs from vertical sections were examined in ImageJ (NIH, Bethesda, Maryland, http://imagej.nih.gov/ij/). The position of the dendritic tips was measured and then expressed as percent vertical penetration relative to the borders of the CtBP2-positive area (‘synaptic region’) across the length of several sections of retinas from at least two different animals per cell type. Experiments were performed in age-matched mice at postnatal weeks 5 (n = 3) and 10 (n = 2). A difference between these two ages was not observed. Areas of *Nrl*^-/-^ mouse specific rosettes were excluded from the measurements. The borders of the synaptic region were defined by the inner (0%) and outer (100%) margins of CtBP2 (ribbon) staining at the location of each dendritic tip. The data failed the Shapiro-Wilk normality test. Therefore, the Kruskal-Wallis one-way ANOVA with post-hoc tests (Dunn’s Multiple Comparison) were applied to determine if cell types were significantly different from each other.

### Electron microscopy

Vibratome sections were incubated for 4 d at 4°C in a primary antibody solution containing 3% normal goat serum (NGS), 1% bovine serum albumin, and 0.05% sodium azide in PBS. After rinsing in PBS, biotinylated goat anti-rabbit IgG or goat anti-mouse IgG was applied (1:100; Vector Labs, Burlingame, USA), and a peroxidase-based enzymatic detection system (Vectastain Elite ABC kit; Vector Labs) was used according to the manufacturer’s instructions to visualize antibody binding. The sections were rinsed again in PBS, subsequently in 0.05 M Tris-HCl, pH 7.6, and then treated with 3,3-diaminobenzidine (DAB; 0.05% in Tris-HCl) with 0.01% H_2_O_2_ for 5–10 min. Afterward, the sections were rinsed in Tris-HCl, subsequently in 0.1 M cacodylate buffer, pH 7.4, and then postfixed in 2.5% glutaraldehyde in cacodylate buffer for 1 h. After several washes in distilled water, the DAB reaction product was silver-intensified by incubating the sections in a solution containing 3% hexamethylene tetramine, 5% silver nitrate, 2,5% borax, 2,5% sodium thiosulfate and 0,05% gold chloride for 10 min at 60°C. Subsequently the sections were postfixed with 0.5% OsO_4_ in cacodylate buffer for 30 min and dehydrated in a graded series of ethanol (30–100%) followed by propylene oxide. They were embedded in Epon and serial ultrathin sections were cut, stained with uranyl acetate and lead citrate, and examined with a Zeiss EM10 electron microscope.

## Results

### Dendritic stratification of OFF bipolar cells in *Nrl*^*-/-*^ mice

**Fig 1:**
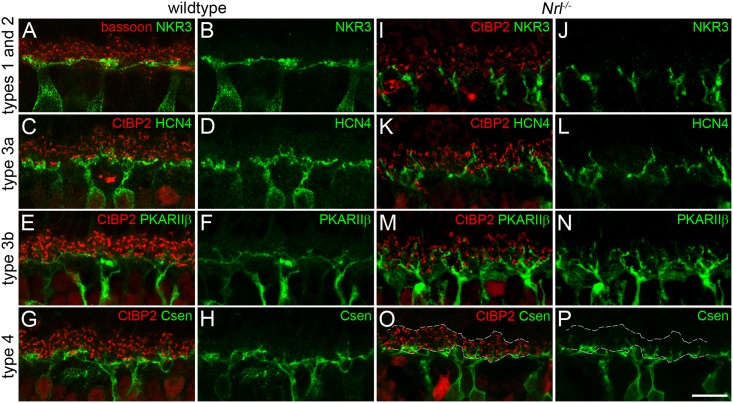
Dendritic stratification patterns of OFF bipolar cells. **A-P:** Maximum intensity projections of confocal image stacks from vertical cryosections of wild-type (A-H) and *Nrl*^-/-^ (I-P) mice double-labeled with ribbon markers bassoon (A) or CtBP2 (all other). OFF bipolar cells were labeled with antibodies against NK3R (types 1 and 2; A,B,I,J), HCN4 (type 3a; C,D,K,L), PKARIIβ (type 3b; E,F,M,N), and calsenilin (Csen, type 4; G,H,O,P). Dashed lines in O indicate the inner and outer border of the CtBP2 labeled area (see Fig 2A for more details). Scale bar = 10 μm for all panels.

The dendritic extension of four different populations of OFF bipolar cells in the mouse retina was investigated relative to the distribution of photoreceptor presynaptic ribbons indicated by bassoon or CtBP2 labeling (‘synaptic region’, Fig 1). OFF bipolar cells were labeled with cell type specific markers (types 1 and 2, NK3R; type 3a, HCN4; type 3b, PKARIIβ; type 4, calsenilin (Csen). As expected, the dendritic tips of all bipolar cell types resided within the innermost quarter of the synaptic region in the wild-type retina, where the cone pedicles are normally positioned (Fig 1A–1H). In the *Nrl*^-/-^ retina, however, the dendrites of all four investigated populations of bipolar cells were found to also extend further into the OPL (Fig 1I–1P). This effect is most evident among type 3b cells whose dendrites often penetrated the whole depth of the synaptic region. Type 1 and 2 cells showed a phenotype similar to the wildtype with only a few dendrites reaching into the outer half of the synaptic region. Type 3a and type 4 cells showed intermediate levels of dendritic extension.

**Fig 2:**
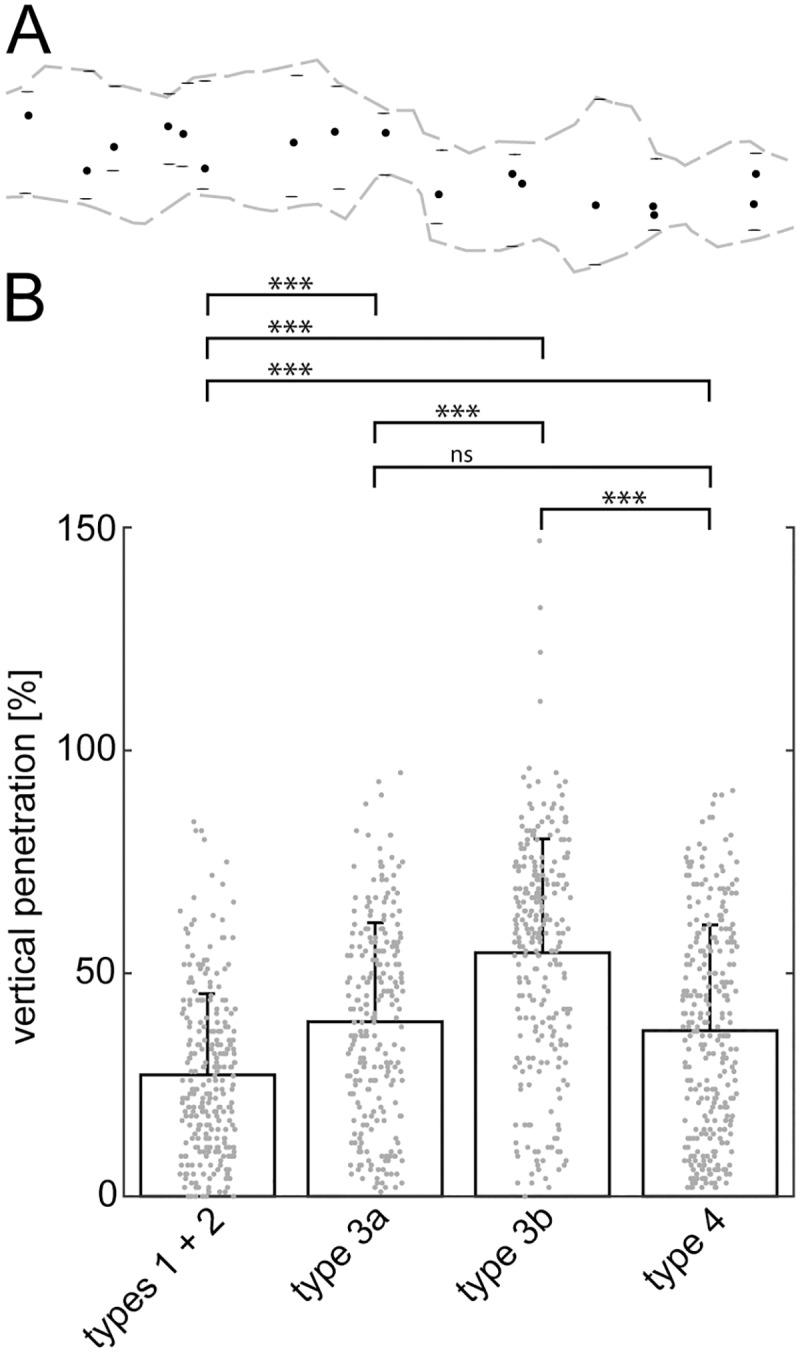
Vertical penetration depth of OFF bipolar cell dendrites through the OPL of *Nrl*^-/-^ retinas. **A:** Schematic showing the outline of the CtBP2-labeled area in the z-projection of Fig 1O (gray dashed lines). Horizontal bars show local borders of the CtBP2-labeled area within the stack where measurements were taken. Vertical penetration depth was measured in single optical sections of the image stacks as the distance between the inner border of the ribbon label (lower horizontal bars) and the location of each dendritic tip (black dot) relative to the whole width of the ribbon label (distance between lower and upper horizontal bar). **B:** Quantification of the extent of dendritic extension through the OPL in *Nrl*^-/-^ mice. Individual measurements are shown as gray dots. The four measurements of values higher than 100% (outer margin of the ribbon label) in case of type 3b OFF bipolar cells occur due to extension of dendrites into the ONL. The bar plots show the mean penetration depth, error bars indicate the standard deviation from the mean. *** indicates significant differences (p<0.001) between corresponding types as determined by Dunn’s Multiple Comparison post-hoc tests (ns, not significant, p>0.05).

These findings were quantified by measuring the positions of dendritic tips relative to the local borders of the synaptic region (Fig 2A). CtBP2 was used exclusively for measuring the termination depth of OFF bipolar cell dendrites in *Nrl*^*-/-*^ retinas. The dendritic stratification patterns differed significantly between the analyzed cell types in the *Nrl*^-/-^ retina (Kruskal-Wallis one-way ANOVA, p<0.0001). Fig 2B shows individual data points, means, and s.d. of the analyzed OFF bipolar cell types. The mean penetration depth of NK3R labeled dendrites (n = 267) through the synaptic region was 27±18% (mean ± s.d.). The large majority of dendrites from types 1 and 2 did not grow beyond the inner half of the synaptic region. This stratification pattern was significantly different from all other cell types (p<0.001) where many more dendrites reached further into the outer synaptic region. Dendrites of type 3a cells (n = 238) terminated on average at 39±22% (mean ± s.d). The number of dendritic tips increased toward the center of the synaptic region, where most tips were located. This pattern was significantly different from types 1/2 and 3b (p<0.001), whereas a comparison with type 4 showed no difference (p>0.05). The stratification pattern of type 3b cells (n = 254 dendritic tips, 55±26% penetration depth, mean ± s.d.) was again significantly different from all other cell types (p<0.001). This cell type produced the most prominent dendritic extension. Interestingly, the majority of dendritic tips was found in the outer half of the synaptic region and four tips of the sample even reached into the outer nuclear layer (ONL, >100%). Dendrites of type 4 cells (n = 290) showed an average penetration depth of 37±24% (mean ± s.d.). The number of dendritic tips was highest in the innermost part of the synaptic region and decreased toward the outer margin. As mentioned above, this pattern was not significantly different from that of type3a cells, whereas a significant difference was found when compared to the remaining types 1/2 and 3b (p<0.001).

### Synapse formation between OFF bipolar cells and cones

**Fig 3:**
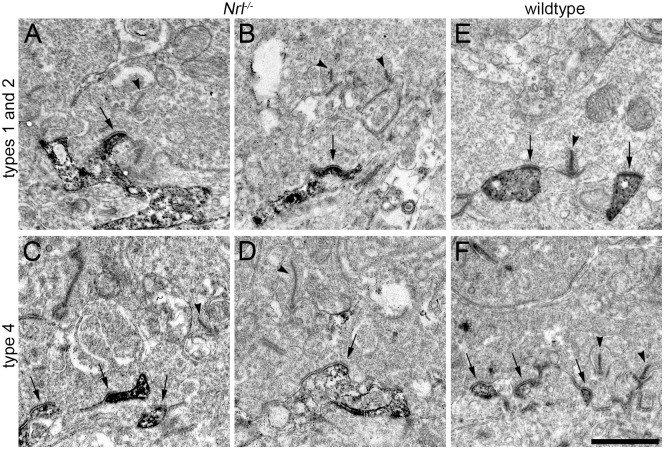
Flat contacts of OFF bipolar cell dendrites with cones. **A-F:** Electron microscopic images of OFF bipolar cell dendrites labeled via pre-embedding immunohistochemistry in vertical sections from *Nrl*^-/-^ (A-D) and wild-type retinas (E,F). Types 1 and 2 bipolar cells were labeled with NK3R (A,B,E), type 4 bipolar cells with calsenilin (Csen, C,D,F). Presynaptic ribbons in cone(-like) photoreceptor terminals are indicated by arrowheads. Putative postsynaptic OFF bipolar cell contacts are marked by arrows where cone membranes appeared thickened and darker than those in surrounding areas, typically indicative of presynaptic contact sites. Scale bar = 1 μm for all panels.

Electron micrographs of immunolabeled ultrathin sections were taken to address the question whether these OFF bipolar cell dendrites extending into the outer reaches of the OPL in *Nrl*^-/-^ retinas form synaptic contacts with photoreceptors (Fig 3A–3D). Although photoreceptor terminals in the *Nrl*^-/-^ mouse did not show the typical cone pedicle-like appearance as in wildtype [22], their characteristic intracellular components (e.g. vesicles, presynaptic ribbons) were readily identified. OFF bipolar cell contacts with photoreceptor terminals consisted of the flat, basal type similar to the wildtype (Fig 3E and 3F) suggesting that synaptic connections were formed.

**Fig 4:**
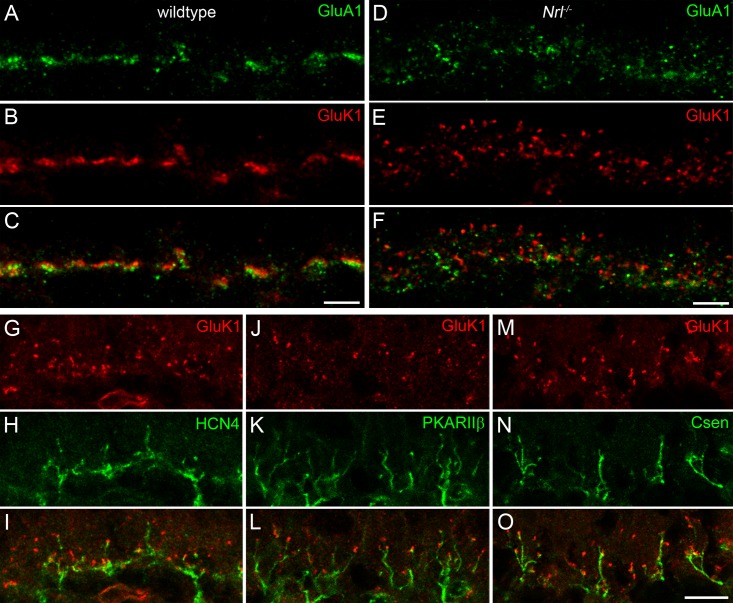
Glutamate receptor expression in the *Nrl*^-/-^ mouse. **A-F:** Maximum intensity projections of confocal image stacks from vertical cryosections of wild-type (A-C) and *Nrl*^-/-^ (D-F) mice double-labeled with GluA1 and GluK1. The typical band-like clusters of GluR immunoreactivity at wild-type cone pedicles does not occur in the *Nrl*^-/-^ mouse, where the GluR labeling shows a punctate spread throughout the whole synaptic region where cone terminals are located. Scale bars = 5 μm. **G-O:** Single optical sections of confocal image stacks from *Nrl*^-/-^ mice double labeled with GluK1 and HCN4 (G-I), PKARIIβ (J-L), and Csen (M-O), respectively. Dendritic tips of type 3a, type 3b, and type 4 OFF bipolar cells are immunoreactive for GluK1. Scale bar = 5 μm for panels G-O.

Successful immunolabeling with bipolar cell markers in electron microscopic specimens were only achieved with markers for types 1/2 (NK3R) and type 4 cells (Csen). To obtain a more complete picture of putative synapse formation between photoreceptors and OFF bipolar cells in *Nrl*^-/-^ mice, dendritic glutamate receptors (GluRs) were labeled. The dominant receptor subunits were previously shown to be GluA1 (AMPA) and GluK1 (kainate) [29]. Double-labeling of GluA1 and GluK1 in the wild-type mouse mainly appears as distinct bands corresponding to flat contacts of OFF bipolar cells at cone pedicles ([29–31] and our study, Fig 4A–4C). The same experiment was performed in *Nrl*^-/-^ mice, where the two GluR subunits were no longer clustered as bands. The labeling occurred as individual puncta and showed a much broader vertical spread throughout the OPL (Fig 4D–4F) corresponding to the expanded synaptic region indicated by CtBP2 labeling in Fig 1. OFF bipolar cell types 3a, 3b, and 4 have been shown to express kainate receptors [29, 32, 33]. In line with the previous finding in wild-type mouse retina, double-labeling of those types with GluK1 in *Nrl*^-/-^ mice revealed that their dendritic tips were decorated with the corresponding receptor subunit (Fig 4G–4O). Taken together, we conclude that these OFF bipolar cell dendrites form synapses with photoreceptor terminals in the *Nrl*^-/-^ mouse.

## Discussion

To analyze dendritic stratification patterns of OFF bipolar cells, we took advantage of the *Nrl*^*-/-*^ mouse retina where all photoreceptor precursor cells develop as cone photoreceptors. Instead of a single row of cone terminals as in wild-type retinas, the synaptic region of cone terminals is greatly increased across the depth of the OPL [22]. This in turn leads to an increased dendritic extension from horizontal cells into the OPL [23], whereas type 7 ON cone bipolar cells largely avoid extending their dendrites into the outer region of the OPL [24]. Here, the structure of the OPL in *Nrl*^*-/-*^ mouse retinas allowed a quantification of the vertical extension of OFF bipolar cell dendrites into the outer parts of the OPL. Our experiments revealed that the stratification patterns of OFF bipolar cells differ significantly between cell types in *Nrl*^*-/-*^ retinas and that they form putative synapses with cone terminals in these outer portions of the OPL.

Interestingly, OFF bipolar cell types 3a, 3b, and 4 have previously been shown to contact rods as well as cones [5, 6, 8] and these were the types which exhibited strong dendritic extension into the OPL in our study. In contrast, cone-selective OFF bipolar cell types 1 and 2 showed only minor extents of dendritic expansion throughout the synaptic region of cone terminals, similar to type 7 ON cone bipolar cells [24]. Our results are consistent with the results of Haverkamp and co-workers [5] who reported a similar behavior of OFF bipolar cells in the bassoon mutant mouse retina, where synaptic transmission from photoreceptors is disturbed. There, most dendrites of type 1 and 2 OFF bipolar cells also remained at the inner border of the OPL while type 3b and 4 cell dendrites extended into the ONL.

One possible explanation for the distinctive behavior of OFF bipolar cell dendrites is that those differences reflect distinct times of differentiation and colonization of cone pedicles [34, 35]. It may be that types 1 and 2 extend their dendrites in search of target cone pedicles earlier during development, ceasing their foraging for synaptic contacts as soon as their presynaptic partners are contacted. In contrast, the remaining, later-differentiating, types send their dendrites further through the synaptic region, due to the earlier occupancy by the type 1 and 2 dendrites.

Yet another possibility is that this distinctive patterning occurs for exclusively cell-intrinsic reasons, independent of any spatial or temporal variation in presynaptic specializations associated with differences between rods and cones (this study), or when synaptic transmission is strongly attenuated [5]. By this view, intrinsic differences guide dendritic outgrowth in a type-dependent manner, and those differences that permit connectivity with rods in wildtype (i.e. by growing into that portion containing only rod terminals) support the formation of connectivity with cone pedicles now present in the outer parts of the OPL in *Nrl*^*-/-*^ retinas. In line with this hypothesis, a comparison of dendritic connectivity patterns from two ON bipolar cells during retinal development has shown that different strategies (targeted or exploratory) are employed to form contacts with cone photoreceptors [36]. Still to be explained, however, by this account is why some of the OFF bipolar cell types arborize more extensively in the outer parts of the OPL.

Furthermore, our findings directly relate to the cells’ dendritic connectivity patterns in wild-type retina. A quantitative analysis of rod and cone contacts among mouse OFF bipolar cells via reconstruction in a serial block-face electron microscopic sample [15] has revealed that the relative amounts of rod contacts per OFF bipolar cell type correlate with the different expansion levels found in our study (e.g. type 3b cells show strongest dendritic extension in *Nrl*^*-/-*^ and the highest amount of rod contacts in wildtype).

It remains a challenge for future studies to reveal if this differential extension of OFF bipolar cell dendrites is regulated via intrinsic mechanisms which act differently depending on cell type or if the timing of synaptogenesis is the key factor for the type-specific differences observed here. Nevertheless, the consistency of the cells’ distinct dendritic stratification and connectivity patterns between cell types in the aforementioned mouse models suggests that the rod contacts of certain OFF bipolar cell types are facilitated via individual levels of enhanced dendritic extension into the OPL that is independent of any extrinsic signal offered by the rod terminals.
